# Fibroblast growth factor 23 and kidney function in patients with type 1 diabetes

**DOI:** 10.1371/journal.pone.0274182

**Published:** 2022-09-09

**Authors:** Yuichi Takashi, Yasutaka Maeda, Kyoko Toyokawa, Naoki Oda, Rie Yoshioka, Dan Sekiguchi, Masae Minami, Daiji Kawanami

**Affiliations:** 1 Department of Endocrinology and Diabetes Mellitus, Fukuoka University School of Medicine, Fukuoka, Japan; 2 MINAMI Diabetes Clinical Research Center, Clinic Masae Minami, Fukuoka, Japan; Augusta University, UNITED STATES

## Abstract

Diabetic kidney disease (DKD) is a key determinant of morbidity and mortality in patients with type 1 diabetes (T1D). Identifying factors associated with early glomerular filtration rate (GFR) decline in T1D is important in prevention or early intervention for DKD. This study investigated whether phosphate metabolism, including fibroblast growth factor 23 (FGF23) is associated with the kidney function of patients with T1D. We randomly recruited 118 patients with T1D with a normal or mildly impaired kidney function [chronic kidney disease (CKD) stages of G1/G2, A1/A2], and measured their serum FGF23 levels. Serum FGF23 was significantly negatively associated with the estimated GFR (eGFR) (*r* = -0.292, *P* = 0.0016), but not urinary albumin creatinine ratio (UACR), and positively associated with serum phosphate (Pi; *r* = 0.273, *P* = 0.0027). Serum FGF23 increased with decreasing eGFR quartiles (*P* for linear trend = 0.0371), while FGF23 was modestly higher in the higher quartiles of UACR (not statistically significant). The multiple linear regression analysis also showed a significant inverse association between FGF23 and eGFR (Model 1: *β* = -0.149, *P* = 0.0429; Model 2: *β* = -0.141, *P* = 0.0370). The association remained significant after adjustment for Pi. We identified that FGF23 was inversely associated with the eGFR in T1D patients with a normal or mildly impaired kidney function.

## Introduction

Diabetic kidney disease (DKD) is a key determinant of morbidity and mortality in patients with type 1 diabetes (T1D), in whom it causes end-stage renal disease (ESRD) and several types of cardiovascular disease [1, 2]. While typical DKD is characterized by progressive increases in urinary albumin excretion and subsequent declines in the glomerular filtration rate (GFR), some populations show direct declines in their GFR without albuminuria [3–5]. Although the mechanisms underlying how GFR decline precedes the onset of albuminuria remain unclear, it has been proposed that tubular injury can trigger glomerulosclerosis [6], suggesting that the development of DKD does not always begin in the glomerulus. However, it is not clear what damages the tubules from an early-stage. Furthermore, a clinical marker that reflects kidney injury in T1D patients with early-stage (G1/G2, A1/A2) chronic kidney disease (CKD) has not been established. Therefore, identifying a factor that is associated with early GFR decline in T1D is critically important in prevention or early intervention for DKD.

It was reported that higher serum phosphate also accelerates the progression of CKD by promoting kidney aging [7–9]. Phosphate is an essential nutrient for humans and plays many roles in the body [10]. In addition, phosphate is included in almost all foods and is ingested both as a natural component and as a food additive [11]. Thus, the intake of phosphate is considered to be associated with dietary habits. In recent times, due to the higher consumption of processed food, concerns have arisen regarding the possibility that chronic high consumption of phosphate may be toxic for the kidney [12, 13]. Of course, hyperphosphatemia is harmful for the body and is well known to induce vascular calcification, resulting in ischemic heart disease and stroke, especially in patients with ESRD who are on hemodialysis [14]. On the other hand, the impact of phosphate metabolism in T1D patients with a normal or mildly impaired kidney function has not been elucidated.

Fibroblast growth factor 23 (FGF23) is a principal hormone in the regulation of serum phosphate [15, 16]. FGF23 produced by the bone, especially by osteoblasts and osteocytes, can bind to FGF receptor 1c (FGFR1c) and α-Klotho complex in the kidney, and works to reduce the serum phosphate level [14]. FGF23 reduces phosphate reabsorption by suppressing the expression of type II a and II c sodium-phosphate cotransporters in the renal proximal tubules [15]. In addition, FGF23 also suppresses the expression of *CYP27B1*, which encodes 25-hydroxyvitamin D-1α-hydroxylase and enhances that of *CYP24A1*, which produces 25-hydroxyvitamin D-24-hydroxylase. FGF23 works to reduce the serum 1,25-dihydroxyvitamin D [1,25(OH)_2_D] level, which enhances intestinal phosphate absorption [15]. Thus, FGF23 reduces the serum phosphate level by inhibiting both proximal tubular phosphate reabsorption and intestinal phosphate absorption. Therefore, it could be considered that FGF23 provides protection against hyperphosphatemia due to oral phosphate loading.

In fact, a high phosphate diet has been shown to increase the serum FGF23 level in humans [17, 18]. Previously, we showed that a high phosphate diet also increased serum the FGF23 level and renal phosphate excretion via FGFR1 in bone using intact animals [19]. Moreover, it was reported that a higher circulating FGF23 is associated with increased cardiovascular morbidity and a higher risk of mortality in type 2 diabetes (T2D) patients with a normal or mildly impaired kidney function [20], despite the precise mechanisms remaining unclear. In addition, we previously indicated that resistin, which mainly regulates insulin resistance, is positively associated with serum FGF23 levels in patients with T2D [21]. Several studies have shown that diabetes is associated with higher serum FGF23 [22–24], whereas other studies did not find an association [25, 26]. The impact of insulin deficiency and insulin resistance on FGF23 is complicated. In addition, many factors are reported to affect serum FGF23 levels in patients with diabetes, including—but not limited to—serum phosphate, inflammation, early tubular injury, insulin, and oral glucose loading [27]. However, few studies have investigated the clinical significance of serum FGF23 in patients with T1D, especially in relation to their kidney function. In this study, we attempted to investigate whether FGF23 is associated with the kidney function in patients with T1D.

## Subjects and methods

### Study design and population

We randomly recruited 118 patients with T1D with a normal or mildly impaired kidney function (CKD stages G1/G2, A1/A2). Each patient provided their written informed consent. All participants were ≥20 years of age. The mean age was 47.0 years, and 43 males (36.4%) and 75 females (63.6%) participated. All patients were treated by diabetologists and were receiving insulin injections at least four times daily or by continuous subcutaneous insulin infusion. The dose of injected insulin was investigated for at least three days and defined as the average amount of insulin. In addition, participants with macroalbuminuria, anemia, malignancy, infection, eating disorders, and pregnancy were excluded in this study. Furthermore, all were free from the treatment with anti-osteoporotic agents, including vitamin D agents, which interact with the serum level of FGF23.

### Biochemical assay

The following information was extracted from electronic medical records: age, sex, duration of diabetes, body mass index (BMI), systolic blood pressure (SBP), diastolic blood pressure (DBP), and total daily dose of insulin (TDD). Blood and urine samples were obtained during routine medical checkups in hospitals. The glycated hemoglobin (HbA1c) level and the serum levels of creatinine (Cr), total-cholesterol, low-density lipoprotein (LDL)-cholesterol, high-density lipoprotein (HDL)-cholesterol, and triglycerides (TG) were collected from electronic medical records. Based on the serum Cr level, the estimated GFR (eGFR) was calculated according to the equation of the Japanese Society of Nephrology:

$$\text{eGFR}\left(\text{ml}/\text{min}/1.73{\text{m}}^{2}\right)=194\times {\text{Cr}}^{-1.094}\times {\text{age}}^{-0.287}(\times 0.739\;\text{if}\;\text{female}).$$

In addition, we measured urinary Cr levels and urinary albumin levels by a colorimetric assay (Cayman chemical, Ann Arbor, USA) and an enzyme-linked immunosorbent (ELISA) assay (Proteintech, Tokyo, Japan), respectively. The serum phosphate (Pi) and urinary Pi levels were examined at a commercial laboratory (SRL, Tokyo, Japan). Moreover, tubular reabsorption of phosphate (TRP) was calculated as follows:

TRP=1–(urinaryPi×serumCr)/(urinaryCr×serumPi).

Finally, we measured serum active full-length FGF23 levels by an ELISA (Kainos, Tokyo, Japan) [28].

### Statistical analysis

Continuous variables with normal or non-normal distributions were described as the mean ± standard error (SE) or the median (Q1, Q3), respectively. Because the duration of diabetes, TDD, body weight-adjusted TDD (TDD/kg), urinary albumin creatinine ration (UACR), total-cholesterol, LDL-cholesterol, TG, TRP, and FGF23 did not show normal distributions, the data were analyzed using natural logarithmic transformed values. Pearson’s correlation coefficient and the Jonckheere-Terpstra trend test were used. To confirm the association between FGF23 and eGFR, we performed a multiple linear regression analysis adjusted for age, sex, BMI, and HbA1c as Model 1 and an analysis with forward-backward stepwise selection method further adjusted for Pi, TG, TDD/kg, SBP, UACR and TRP (in addition to Model 1) as a Model 2. *P* values of <0.05 were considered to indicate statistical significance. Statistical analyses were performed using the BellCurve 3.21 software program (SSRI, Tokyo, Japan).

### Ethical approval

This study was approved by the research ethics committees of both Fukuoka University School of Medicine (#U20-06-007) and Clinic Masae Minami (#MERC-20-003).

## Results

### Clinical characteristics of the patients with T1D

**Fig 1:**
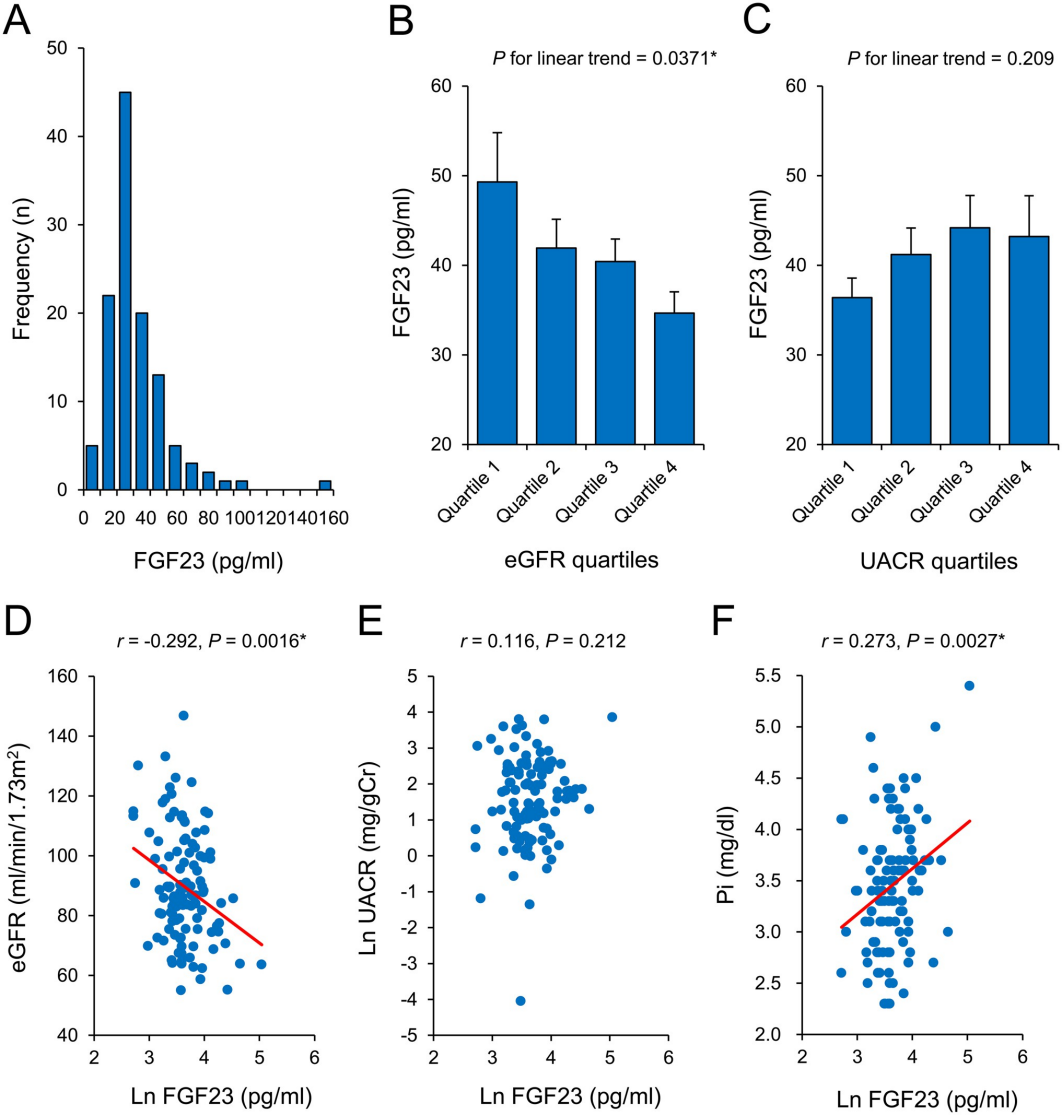
Serum FGF23 was negatively associated with the eGFR, but not the UACR, in patients with T1D. (A) The distribution of serum full-length FGF23 levels. The median (Q1, Q3) serum FGF23 level was 36.6 (30.7, 47.1) pg/ml. (B and C) Descending quartiles of the eGFR were associated with significantly increased mean (±SE) serum FGF23 levels (*P* for linear trend = 0.0371) (B). On the other hand, ascending quartiles of UACR were not significantly associated with increased mean (±SE) serum FGF23 level (*P* for linear trend = 0.209) (C). The Jonckheere-Terpstra trend test was used in these analyses. (D-F) The associations between the serum FGF23 level and the eGFR (*r* = -0.292, *P* = 0.0016) (D), UACR (*r* = 0.116, *P* = 0.212) (E), serum Pi level (*r* = 0.273, *P* = 0.0027) (F). Non-normally distributed variables were subjected to natural logarithmic transformation. Pearson’s correlation coefficients were used in these analyses.

The characteristics of the subjects in this study are shown in Table 1. The mean (±SE) BMI and HbA1c were 23.1±0.3 kg/m^2^ and 7.9±0.08%, respectively. The median duration of diabetes was 14.0 years, TDD was 35.0 units, and TDD/kg was 0.58 units/kg. The mean SBP was 115.1±1.3 mmHg and DBP was 65.8±0.8 mmHg. The mean Cr level was 0.65±0.01 mg/dl, the mean eGFR was 89.8±1.7 ml/min/1.73m^2^, and the median UACR was 5.0 mg/gCr. The mean or median total-cholesterol, LDL-cholesterol, HDL-cholesterol and TG levels were 191 mg/dl, 102 mg/dl, 72.6±1.6 mg/dl, and 85 mg/dl, respectively. Finally, the mean serum Pi level was 3.5±0.06 mg/dl, the median TRP level was 0.91, and the median serum active full-length FGF23 level was 36.6 pg/ml. The distribution of FGF23 in this study is shown in Fig 1A. Taken together, the participants showed a normal kidney function without a decline in the eGFR or albuminuria, and—on the whole—their serum Pi and FGF23 levels were within the reference range.

**Table 1:** Clinical characteristics of the subjects.

	Mean ± SE, median (Q1, Q3) or number (%)
Age (years)	47.0 ± 1.4
Sex (male, female)	43 (36.4%), 75 (63.6%)
Duration of diabetes (years)	14.0 (8.2, 22.8)
BMI (kg/m^2^)	23.1 ± 0.3
SBP (mmHg)	115.1 ± 1.3
DBP (mmHg)	65.8 ± 0.8
TDD (units)	35.0 (26.1, 44.0)
TDD/kg (units/kg)	0.58 (0.45, 0.69)
HbA1c (%)	7.9 ± 0.08
Creatinine (mg/dl)	0.65 ± 0.01
eGFR (ml/min/1.73m^2^)	89.8 ± 1.7
UACR (mg/gCr)	5.0 (2.2, 11.8)
Total-cholesterol (mg/dl)	191 (175, 215)
LDL-cholesterol (mg/dl)	102 (88, 120)
HDL-cholesterol (mg/dl)	72.6 ± 1.6
TG (mg/dl)	85 (56.3, 111.3)
Pi (mg/dl)	3.5 ± 0.06
TRP	0.91 (0.86, 0.94)
FGF23 (pg/ml)	36.6 (30.7, 47.1)

Continuous variables with normal or non-normal distributions were described as the mean ± standard error (SE) or the median (Q1, Q3), respectively.

BMI, body mass index; SBP, systolic blood pressure; DBP, diastolic blood pressure; TDD, total daily dose of insulin; TDD/kg, body weight-adjusted TDD; HbA1c, hemoglobin A1c; eGFR, estimated glomerular filtration rate; UACR, urinary albumin creatinine ratio; TG, triglycerides; Pi, inorganic phosphate; TRP, tubular reabsorption of phosphate; FGF23, fibroblast growth factor 23

### The associations between FGF23 and each parameter in patients with T1D

The serum FGF23 levels of subjects were significantly and positively correlated with age (*r* = 0.263, *P* = 0.0041), Cr (*r* = 0.294, *P* = 0.0015), TG (*r* = 0.210, *P* = 0.025) and serum Pi level (*r* = 0.273, *P* = 0.0027), and negatively correlated with the eGFR (*r* = -0.292, *P* = 0.0016) (Table 2). On the other hand, FGF23 was not correlated with the duration of diabetes (*r* = 0.158, *P* = 0.088), BMI (*r* = 0.171, *P* = 0.064), SBP (*r* = 0.125, *P* = 0.176), DBP (*r* = 0.132, *P* = 0.156), TDD (*r* = 0.048, *P* = 0.608), TDD/kg (*r* = -0.006, *P =* 0.948), HbA1c (*r* = -0.172, *P* = 0.064), UACR (*r* = 0.116, *P* = 0.212), total-cholesterol (*r* = -0.010, *P* = 0.920), LDL-cholesterol (*r* = -0.101, *P* = 0.285), HDL-cholesterol (*r* = -0.084, *P* = 0.377), or TRP (*r* = -0.101, *P* = 0.283) (Table 2).

**Table 2:** Associations between FGF23 and each parameter.

	*r*	*P* value
Age (years)	0.263	0.0041*
Duration of diabetes (years)	0.158	0.088
BMI (kg/m^2^)	0.171	0.064
SBP (mmHg)	0.125	0.176
DBP (mmHg)	0.132	0.156
TDD (units)	0.048	0.608
TDD/kg (units/kg)	-0.006	0.948
HbA1c (%)	-0.172	0.064
Creatinine (mg/dl)	0.294	0.0015*
eGFR (ml/min/1.73m^2^)	-0.292	0.0016*
UACR (mg/gCr)	0.116	0.212
Total-cholesterol (mg/dl)	-0.010	0.920
LDL-cholesterol (mg/dl)	-0.101	0.285
HDL-cholesterol (mg/dl)	-0.084	0.377
TG (mg/dl)	0.210	0.025*
Pi (mg/dl)	0.273	0.0027*
TRP	-0.101	0.283

The Pearson’s correlation coefficient was used.

* Statistically significant (*P* < 0.05)

FGF23, fibroblast growth factor 23; BMI, body mass index; SBP, systolic blood pressure; DBP, diastolic blood pressure; TDD, total daily dose of insulin; TDD/kg, body weight-adjusted TDD; HbA1c, hemoglobin A1c; eGFR, estimated glomerular filtration rate; UACR, urinary albumin creatinine ratio; TG, triglycerides; Pi, inorganic phosphate; TRP, tubular reabsorption of phosphate.

### The association between FGF23 and the kidney function in patients with T1D

The serum FGF23 level increased with decreasing eGFR quartiles (*P* for linear trend = 0.0371) (Fig 1B), while FGF23 was modestly higher in the higher quartiles of UACR (statistically not significant, *P* for linear trend = 0.209) (Fig 1C). As mentioned above, the serum FGF23 level showed a significant negative association with the eGFR (Fig 1D), but not the UACR (Fig 1E). In addition, the serum FGF23 level was also positively associated with the serum Pi level (Fig 1F).

Then, the multiple linear regression analysis adjusted for age, sex, BMI, and HbA1c also showed a significant association between FGF23 and eGFR (*β* = -0.149, *P* = 0.0429) (Table 3: Model 1). In the univariate analyses, FGF23 was significantly correlated with age, eGFR, TG, and Pi, as shown in Table 2. On the other hand, the eGFR was significantly correlated with TDD/kg, SBP, UACR, and TRP. Then, we further performed multiple regression analyses adjusted for these parameters. As a result, the association between FGF23 and eGFR remained significant after further adjustment for Pi (*β* = -0.158, *P* = 0.0448), TG (*β* = -0.171, *P* = 0.0182), TDD/kg (*β* = -0.154, *P* = 0.0371), and SBP (*β* = -0.146, *P* = 0.0481) (Table 3). However, this association did not remain after adjustment for the UACR (*β* = -0.122, *P* = 0.1004) and TRP (*β* = -0.142, *P* = 0.0506) (Table 3). Furthermore, the multiple linear regression analysis with forward-backward stepwise selection adjusted for all of these parameters, in addition to those in a Model 1, also showed the significant association between FGF23 and the eGFR (*β* = -0.141, *P* = 0.0370) (Table 3: Model 2).

**Table 3:** Association between FGF23 and eGFR in patients with type 1 diabetes.

	Standardised *β*	*P* value
Univariate model	-0.292	0.0016*
(adjusted R^2^ = 0.085)		
Multivariate model		
Model 1 (adjusted R^2^ = 0.502)	-0.149	0.0429*
Model 1 + Pi (adjusted R^2^ = 0.497)	-0.158	0.0448*
Model 1 + TG (adjusted R^2^ = 0.527)	-0.171	0.0182*
Model 1 + TDD/kg (adjusted R^2^ = 0.502)	-0.154	0.0371*
Model 1 + SBP (adjusted R^2^ = 0.498)	-0.146	0.0481*
Model 1 + UACR (adjusted R^2^ = 0.510)	-0.122	0.1004
Model 1 + TRP (adjusted R^2^ = 0.515)	-0.142	0.0506
Model 2 (adjusted R^2^ = 0.555)	-0.141	0.0370*

Model 1: The multiple regression analysis adjusted for sex, age, BMI, and HbA1c

Model 2: The multiple regression analysis with forward-backward stepwise selection method further adjusted for Pi, TG, TDD/kg, SBP, UACR and TRP in addition to a Model 1

* Statistically significant (*P* < 0.05)

FGF23, fibroblast growth factor 23; eGFR, estimated glomerular filtration rate; BMI, body mass index; HbA1c, hemoglobin A1c; Pi, inorganic phosphate; TG, triglycerides; TDD/kg, body weight-adjusted total daily dose of insulin; SBP, systolic blood pressure; UACR, urinary albumin creatinine ratio; TRP, tubular reabsorption of phosphate.

## Discussion

The main finding of this study is that serum FGF23 level is negatively associated with the eGFR in T1D patients with a normal or mildly impaired kidney function (3 patients with eGFR<60 ml/min/1.73m^2^ and 7 patients with UACR>30 mg/gCr). It is known that the serum FGF23 level starts to increase early in the progression of CKD (eGFR<75 ml/min/1.73m^2^), before the increase of the serum Pi level [29, 30]. This increase in FGF23 was considered to be associated with enhanced urinary phosphate excretion [31]. Generally, a high phosphate condition results in cardiovascular morbidity in patients with advanced DKD [14]. However, the impact of phosphate metabolism on early-stage DKD is unclear.

Multiple prior studies have shown an association between higher FGF23 and subsequent risk of developing ESRD [32–34]. On the other hand, there are less data on the association between FGF23 and early kidney related outcomes. Drew et al. reported that FGF23 is a biomarker of kidney function, however, higher FGF23 is not consistently associated with decline in kidney function or incident CKD in community-dwelling older adults with well-preserved kidney function [35]. Concerning DKD, a case control study of patients enrolled in the Action to Control Cardiovascular Risk in Diabetes (ACCORD) Trial demonstrated that baseline serum FGF23 levels did not predict the CKD incidence in 590 T2D patients [36]. Importantly, FGF23 has been implicated as an independent risk factor for the progression of CKD in T2D patients with microalbuminuria [37]. In that study, baseline serum FGF23 levels were not associated with serum levels of calcium or Pi, or 24-hour urinary Pi excretion. However, increased serum FGF23 levels were shown to be associated with a higher risk of the composite renal outcome, defined as death, doubling of serum creatinine, and/or dialysis [37]. Thus, the significance of FGF23 as a predictive marker for DKD is not constant in T2D. Furthermore, the relationship between FGF23 and eGFR decline in patients with T1D remains unclear. In the present study, we demonstrate—for the first time—that serum FGF23 levels are negatively associated with the eGFR in T1D patients.

Because FGF23 is a phosphotropic hormone, the positive association between serum FGF23 and Pi is reasonable, even though both the serum Pi and FGF23 levels were within the reference range. While a high dietary phosphate intake increases the serum FGF23 level and renal phosphate excretion, the serum Pi level is only slightly increased in intact animals [19]. It seems to prevent the development of hyperphosphatemia—at least in part—by enhancing urinary phosphate excretion via FGF23. Increased serum FGF23 levels are considered to be caused by excessive phosphate intake due to dietary habits. Because phosphate is ingested as a natural component from all types of food and as a food additive, dietary phosphate control is quite difficult in the real-life setting [11]. This could explain—at least in part—the association between FGF23 and TG. In support of this concept, high serum Pi levels have been shown to be associated with increased TG levels in patients with T2D [38]. On the other hand, the serum FGF23 level was not associated with TRP in this study. Due to the use of spot urine tests, it is possible that TRP did not precisely reflect the renal phosphate excretion of the patients. Previous studies also indicated the complexity of the association between FGF23 and urinary phosphate excretion in CKD patients [37, 39]. However, the association between FGF23 and the eGFR became non-significant after additional adjustment for TRP. This suggested that TRP affected—at least in part—the association between FGF23 and the eGFR in this study. While FGF23 enhances phosphate excretion in the renal proximal tubules, the eGFR was associated with FGF23, but not the UACR, in this study. Thus, we can speculate that dietary phosphate loading and high FGF23 condition—even in normophosphatemia—induce hyperphosphaturia, which triggers proximal tubular injury and the initial progression of the eGFR decline without albuminuria and glomerular injury in T1D patients who have susceptibility to renal damage [40]. On the other hand, the association between FGF23 and the eGFR also became non-significant after additional adjustment for the UACR. These results suggested that glomerular injury also affects the association between FGF23 and the eGFR. It has been demonstrated that tubular damage can trigger glomerulosclerosis [6, 41]. A high phosphate diet has been implicated as a driver of kidney aging mainly by enhancing tubular senescence and tubulointerstitial fibrosis [42], suggesting that the first place to be damaged by phosphate is the tubules and that glomerulosclerosis develops subsequently. As most patients in our study were normoalbuminuric, it was difficult to conclude the relationship between FGF23 and the degree of albuminuria. Further studies are needed to clarify the detailed mechanism underlying the relationship between DKD and phosphate metabolism, including FGF23.

The impact of insulin deficiency as T1D on FGF23 is complicated. A recent report indicated that insulin suppresses the production of FGF23 in the bone [43]. In addition, it has been shown that FGF23 and insulin have an inverse correlation in healthy subjects [43]. On the contrary, insulin resistance is significantly associated with the increase of FGF23 [23]. These findings indicate that hyperinsulinemia inhibits the production of FGF23. Therefore, we hypothesized that TDD or TDD/kg could be factors affecting the serum FGF23 levels in patients with T1D. However, there were no associations between FGF23 and TDD or TDD/kg. Although the precise mechanisms underlying this observation are unclear, Dogan et al. reported that there was no difference in the serum FGF23 levels of T1D patients and healthy individuals, but that FGF23 was related to cardiac diastolic dysfunction in T1D patients with early DKD [44]. Taken together, it seems that FGF23 may reflect cardiorenal injury independent of exogenous insulin, at least, in T1D. Various factors, such as phosphate intake and inflammation, have been shown to modify serum FGF23 levels in patients with diabetes [27]. Among these factors, we considered that phosphate is a main inducer of the production of FGF23, because FGF23 is a phosphotropic hormone. Previously, we reported that phosphate could directly activate FGFR1 independent of FGFR ligands and that downstream signal transduction could increase the active full-length FGF23 level through the gene product of *GALNT3* [19].

The present study was associated with some limitations. First, the conclusions of this study are weakened by the cross-sectional design. Further longitudinal studies are required to explore the causal directions. Second, the sample size was small and all participants were Japanese. Third, we did not evaluate any clinical markers of renal tubular injury, or the serum level of 1,25(OH)_2_D and parathyroid hormone (PTH), which are associated with serum FGF23 levels. Fourth, we could not simultaneously compare subjects without diabetes or patients with T2D.

In conclusion, we identified that FGF23 was negatively associated with the eGFR in T1D patients with a normal or mildly impaired kidney function. Our study provides a rationale for future prospective studies to investigate whether FGF23 is associated with the early progression of DKD in patients with T1D.

## Supporting information

S1 File (XLSX)
